# Mentes Positivas en Acción: A Randomized Feasibility Study of a Promotor-Delivered Cognitive Behavioral Stress Management Program for Low-Income Spanish-Speaking Latinos

**DOI:** 10.1089/heq.2020.0092

**Published:** 2021-04-21

**Authors:** Rosa María Sternberg, Anita L. Stewart, Anna María Nápoles

**Affiliations:** ^1^ Center for Aging in Diverse Communities, Institute for Health & Aging, University of California San Francisco (UCSF), San Francisco, California, USA.; ^2^ Department of Family Health Care Nursing, UCSF School of Nursing, San Francisco, California, USA.; ^3^ Division of Intramural Research, National Institute on Minority Health and Health Disparities, National Institutes of Health, Bethesda, Maryland, USA.

**Keywords:** Latino stress, promotores, community interventions, mental health care access

## Abstract

**Purpose:** Although psychological distress is common among Latinos in the United States, they underutilize mental health services. We describe a community-based program to manage stress and reduce depressive symptoms among low-income Spanish-speaking Latinos.

**Methods:** *Mentes Positivas en Acción* (MPA) (*Positive Minds in Action*) is an 8-week group program, delivered by trained promotores in community settings and evaluated through a randomized feasibility study. Participants were randomly assigned to an immediate MPA treatment group or a delayed-intervention control group. Outcomes assessed at baseline and 8 weeks included stress (Perceived Stress Scale [PSS]) and depressive symptoms (PHQ-9); higher scores indicate worse health. Repeated-measures analysis of variance examined group×time interaction effects for group differences in change from baseline to 8 weeks. The control group offered the program after the 8-week assessment, completed an additional assessment at the end of the program (16 weeks); *t*-tests assessed within-group changes.

**Results:** Most participants were female, born in Mexico, and spoke only Spanish. Group×time interaction effects were significant for both outcomes. Mean PSS scores improved in the treatment group but not the control group (−0.80 vs. +0.10; *p*<0.014). Mean PHQ-9 scores improved more in the treatment group than the control group (−5.7 vs. −0.3; *p*<0.011). Within-group analyses of the control group found significant improvements in stress (−0.8; *p*<0.000) and depressive symptoms (−3.9; *p*<0.002).

**Conclusions:** This study provides preliminary evidence of the effectiveness of a community-based promotor-delivered program to manage stress and reduce depressive symptoms among vulnerable underserved Latinos in the United States.

## Introduction

In 2018, Latinos constituted 18% of the U. S. population (60 million), and they are projected to make up 28% (111 million) by 2060.^1^ Nearly 20 million Latinos living in the United States are immigrants. With a median age of about 30 years,^2^ compared with 55 years for whites in the United States,^3^ maintaining the health of this young workforce is essential to the socioeconomic future of our country. One area requiring focused attention is promoting their mental health and well-being. Almost half of adult Latinos living in the United States report that their situation has worsened over the past few years.^4,5^ In addition, a worsening anti-immigrant climate in the decade leading up to the 2016 election has been followed by mainstreaming of ideologies against immigrants who were once on the fringes of society.^6^ Anti-immigrant policies contribute to stress and a myriad of psychosocial problems for immigrant Latinos and their families, with job and housing discrimination as major concerns.^4,7–9^ Also, these policies and accompanying discrimination portend greater difficulty in recruiting and retaining immigrant populations in clinical research.^10,11^

Despite their elevated risk for depression, Latinos underutilize mental health services.^12^ Low income, limited English proficiency, lack of health insurance, and cultural barriers are among the leading obstacles that hinder Latinos' ability to access help for stress and depression-related symptoms, especially those who speak primarily Spanish.^13,14^

One approach to increasing access to mental health services is to provide low-cost culturally appropriate interventions in Spanish that can be delivered in community settings.^15^ Promotores (community health workers) are being trained increasingly to deliver community-based interventions, with positive results among communities suffering health disparities.^16,17^ Promotores can easily establish rapport and transmit self-care knowledge and model desired behaviors.^18,19^ Although promotor-delivered mental health interventions can be effective in underserved populations, a few have focused on Latinos.^20,21^

To meet this need, we developed *Mentes Positivas en Acción* (MPA) (*Positive Minds in Action*), which is delivered by trained promotores and aims at managing stress and reducing depressive symptoms in community-dwelling Latinos. We first tested its feasibility in a single-arm pilot study of Spanish-speaking Latinos, observing pre–post intervention reductions in general stress and depressive symptoms.^22^ This article reports results of a randomized feasibility study of MPA, delivered in the community to low-income Spanish-speaking Latinos who indicated that they were feeling stressed or depressed. We also report our experiences of recruiting and retaining Latinos within the worsening environment for immigrants.

## Methods

### Study design

This study was a real-world community-based randomized feasibility trial of the MPA program, conducted between February 2016 and March 2017. The 8-week MPA consists of weekly 2-h group classes of four to six participants, led by two trained promotores. There were two experimental conditions: The treatment group received the MPA program immediately (hereafter referred to as the “treatment group”) and the delayed-intervention control group received the program 8 weeks later, after the 8-week assessment (hereafter referred to as the “control group”). We examined the effectiveness of MPA in reducing stress and depressive symptoms at 8 weeks. After the 8-week assessment, individuals in the control group were offered the program, after which they completed a 16-week assessment to measure changes in outcomes. The University of California San Francisco Institutional Review Board approved the study protocol; written informed consent was obtained from all participants.

### Community partnership

The study was conducted in partnership with Monument Impact, a nonprofit community-based organization in Concord, California that serves disadvantaged residents through programs promoting mental and physical health and building workforce skills. Monument Impact has a promotores service delivery model and was interested in adding MPA to their programs. Monument Impact provided space for participant screening and intervention delivery.

### Sample and recruitment

Study participants were Spanish-speaking Latinos residing in Concord, CA. Inclusion criteria were: (1) age >18 years, (2) self-identified as primarily Spanish-speaking Latino, and (3) endorsed questions of either: “Are you living with a lot of stress?” or “Are you feeling depressed?” Participants were not required to meet diagnostic criteria for depression or generalized anxiety disorder or a threshold for stress symptoms. Participants were recruited through flyers posted at Monument Impact and other Concord locations, and by word of mouth. Individuals were asked to call Monument Impact for more information, and a trained bilingual, bicultural Research Associate (RA) briefly described the program. The RA explained that if they agreed, they would be randomized to begin the program immediately or wait 8 weeks. Interested individuals were screened for eligibility; those eligible made an in-person appointment where written informed consent was completed (the RA read the contents aloud for those needing help). The consent form indicated that they would receive a $150 gift card for completing the study. Participants then completed the baseline assessment, which took ∼30 min. Each participant was assigned a code number, which was recorded on a card and placed in a box to be randomized later. Thus, they left without knowing their randomization group.

### Randomization

**FIG. 1.**
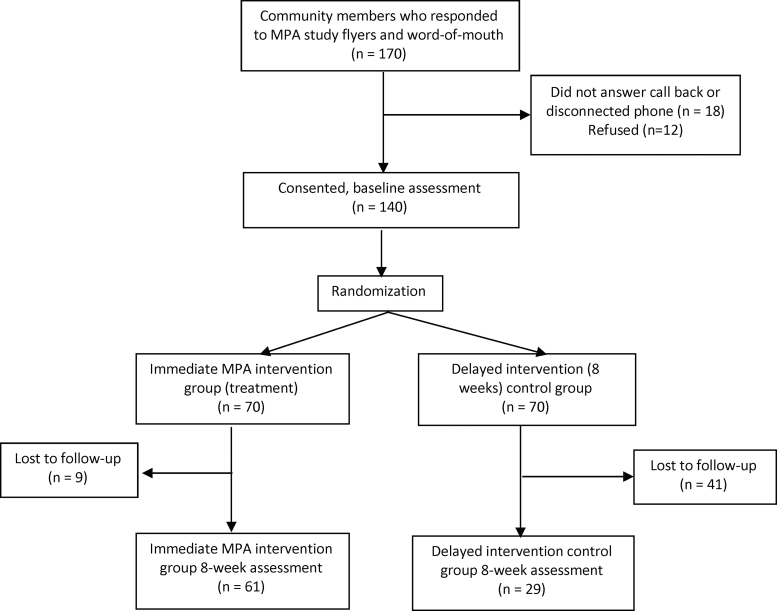
Summary flow chart of MPA participants. MPA, Mentes Positivas en Acción.

Rolling enrollment was used to ensure four to six participants in each group-based MPA program. Once 12 people were enrolled, that group was randomly assigned. Participants' codes were placed in a box and the Principal Investigator randomly (blinded) chose participants to be in the treatment or control group, assuring sufficient participants for each group (Fig. 1). Participants were called and told of their assignment. Participants randomized to the treatment group were assigned to a group program; those in the control group were told that the RA would call them in 7 weeks to begin the MPA program.

### Retention

To maximize retention, promotores made weekly phone calls to participants in the treatment group to remind them of the sessions. For those in the control group, during the seventh week of the waiting period, the RA contacted them to schedule their 8-week assessment and inform them of the program's start date, time, and location.

### Intervention content and format

The 8-week MPA program is a manualized, group-based program to manage stress and reduce depressive symptoms in lower income Spanish-speaking Latinos. It consists of weekly 2-h classes of four to six participants each, led by a team of two promotores. The MPA program development included addressing the stigma of stress and depression and optimal delivery mechanisms.^23^ It is based on an integration of three evidence-based programs designed to teach Latinos skills for preventing and managing stress and depressive symptoms.^16,24,25^ Adaptations to enhance cultural sensitivity included content adaptation, tailoring the language to low literacy, using metaphors, and accommodating participants' work schedules.^22,23^

**FIG. 2.**
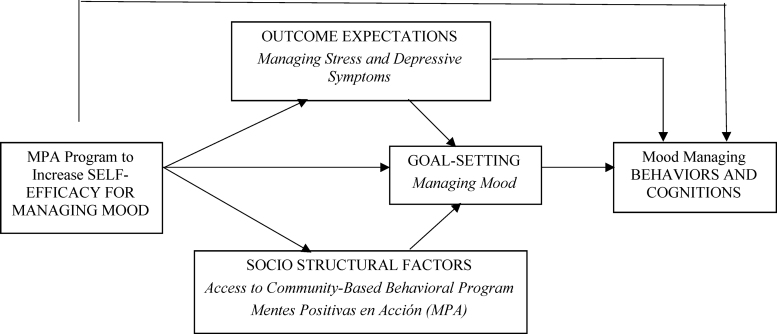
Model of MPA guided by Social Cognitive Theory.

The underlying theoretical approach was the Social Cognitive Theory. The program emphasized cognitive–behavioral coping skills, including mood management, cognitive reframing, increasing pleasant activities, and social skills training, all aimed at increasing self-efficacy for managing mood; the conceptual model is depicted in Figure 2.^25,26^

Weekly sessions took place in Monument Impact's conference room and in other community organizations. Program content included: what is stress and how to manage it, relaxation techniques, personal affirmations, what is depression and how to manage it, how thoughts affect mood, and how to manage negative thoughts. Each week, promotores distributed a packet of that week's material to be inserted in a binder, thus the binder eventually included all program contents. Each session began with a relaxation exercise and homework review. Participants were encouraged to take turns reading and discussing the manual, and to share personal stories related to the program. Each session included a homework assignment (e.g., daily record of mood and how it related to their thoughts). The session ended with participants and promotores sharing expressions of personal gratitude. Based on other stress management programs, we required that participants attend at least four of the eight sessions to be considered as having completed the program and received the core program components.^25,27^

### Promotores recruitment and training

Promotores were recruited through the Monument Impact promotores program and by word of mouth. A total of 16 promotores participated in this study. The majority were female (75%) and born in Mexico. Age ranged from 27 to 67 years (mean=47.2, SD=8.5). All promotores had children, and 68.8% were partnered; 75% reported a family annual income between $20,000 and $50,000. Nearly one-third (31.3%) completed less than 12 years of formal education, and 43.8% completed some college. Their mean years of experience as promotores was 4.7 years (SD=3.7), with a range of 2–16. The MPA promotores' training is detailed elsewhere.^22^ Promotores were required to attend all training sessions and were given $150 on completion of the training and another $150 when they completed the delivery of the MPA program. They were assigned to lead MPA sessions in pairs. The same teams delivered the program to treatment and control group participants and were blinded to treatment group assignment.

### Fidelity of intervention delivery

The PI and RA assessed fidelity of intervention delivery through direct observation of two sessions (selected at random by the PI or RA) of each 8-week MPA group program, including those delivered to treatment and control group participants. They did not announce ahead of time which sessions they would visit. A 16-item structured rating scale was used to rate the extent to which each team delivered the program as intended. Examples of items include the frequency with which the promotores followed the manual, explained concepts in words that participants could understand, guided participants to practice cognitive reframing, and emphasized the homework. Response options were 1=not at all, 2=a fair amount, 3=a great deal, and 4=consistently. After the first of two observed sessions per group program, the observer provided feedback to promotores by using the fidelity ratings. Fidelity scores were calculated at the session and team levels. For each directly observed session, scores from all 16 items were averaged to get a total (overall) fidelity score. To calculate overall fidelity scores for each team, fidelity scores were averaged across all group programs they delivered.

### Assessments

All participants were assessed in Spanish at baseline and 8 weeks. Control group participants who completed the program (8 weeks after starting their program) were assessed at the end (16 weeks after baseline). Most assessments were self-administered, although the RA administered questionnaires to a few low-literacy participants. For practical purposes and since this was a real-world translation study, blinded assessments were not possible.

Perceived stress was measured by using a modified Perceived Stress Scale (PSS).^28^ The modified scale consisted of the seven negatively worded statements (e.g., unable to control important things; felt nervous and stressed; felt that could not cope). Each item is rated from 0 to 4, with 4 indicating high stress. This modification was done to improve its psychometric properties in a study of Mexican immigrant women; the 7-item PSS demonstrated good reliability (Cronbach alpha of 0.82).^22^

To measure depressive symptoms, we used the Spanish version of the PHQ-9 that has been found to have good internal consistency reliability among Latinos (α=0.85).^29,30^ This 9-item self-report measure assesses the frequency of symptoms on a 4-point scale (from 0=not at all to 3=nearly every day). Total scores range from 0 to 27, with higher scores indicating more symptoms. Basic demographic characteristics were assessed, including age, years of education, language, family annual income, and health insurance.

### Data analysis

Independent samples' *t*-tests and chi-square tests of independence were conducted to examine whether there were significant differences among participants at baseline regarding demographic characteristics. Due to significant differences at baseline in participants' partnered status, we controlled for partnered status when testing the results of outcome variables.

We calculated means and standard deviations for perceived stress and depressive symptoms. Using an intention-to-treat approach and repeated-measures analysis of variance, we assessed changes over time (baseline to 8 weeks) in stress and depressive symptoms in the immediate treatment versus control group.

To explore within-group changes in the control group after completing the program, we compared control group scores at the 8-week assessment (after the waiting period, before starting the program) with 16-week scores (after program completion) by using *t*-tests of change scores.

## Results

A total of 170 individuals contacted Monument Impact about the study. Of these, 18 could not be reached by phone. Of the remaining 152 who were contacted, 12 were no longer interested after hearing details about the program and study. Thus, 140 (82%) individuals consented and were enrolled (Fig. 1). Participant characteristics are shown in Table 1. Participants were between 23 and 82 years of age (mean=42.6; SD=14.21). Most were female, born in Mexico, and spoke Spanish only. More than half had a high school education, had children, and were partnered. More than 70% had health insurance and reported a household annual income of <$25,000.

**Table 1.** Baseline demographic characteristics of Mentes Positivas en Acción participants

Characteristic	*n* (%)^a^		*p*
	Treatment (*N*=70)	Control (*N*=70)	
Age, mean (SD)	42.6 (14.2)	42.9 (9.3)	0.930
Range	23–82	30–63	
Gender: female	48 (90.6)	22 (84.6)	0.467
Country of birth			0.168
Mexico	36 (67.9)	22 (84.6)	
United States	5 (9.4)	0 (0)	
Other	12 (22.6)	4 (15.4)	
Language spoken			0.333
English	4 (7.5)	0 (0)	
Spanish	44 (83.0)	24 (92.3)	
English and Spanish	5 (9.4)	2 (7.7)	
Partnered	28 (51.9)	24 (92.3)	<0.000
Children	40 (76.9)	24 (92.3)	0.095
Health insurance (Public)			0.600
Insured	40 (78.4)	19 (73.1)	
Education			0.464
Less than high school	25 (47.2)	10 (38.5)	
High school or higher	28 (52.8)	16 (61.5)	
Annual household income			0.072
Less than $15,000	20 (40.0)	6 (24.0)	
$15,000 to $24,999	18 (36.0)	8 (32.0)	
$25,000 or higher	12 (24.0)	11 (44.0)	

^a^ Percentages based on non-missing values.

SD, standard deviation.

We randomly assigned 70 participants to the treatment group and 70 to the control group. Of these, 61 (87%) treatment group and 29 control group participants (41%) completed the 8-week assessment (Fig. 1). The other 50 participants (9 in the treatment group and 41 in the control group) could not be reached (disconnected phones, phones not in service, or no answer) after 2 attempts.

Treatment group participants' attendance was good; half (*n*=35) attended all eight sessions, and 93% (*n*=65) attended six or more sessions. Among control group participants, all 29 who completed the 8-week assessment agreed to begin the program; more than half (*n*=14) attended all 8 sessions, and 38% (*n*=10) attended 6 or more sessions. At baseline, there were no differences in demographic characteristics between treatment and control groups, except partnered status (*p*<0.0001), with control group participants more likely to be partnered (Table 1). There were no significant group differences on baseline measures of stress and depression. At baseline, the mean stress (PSS) score for the treatment group was 2.1 (SD=1.1) and 2.4 (SD=0.8) for the control group; depression scores were 7.8 (SD=6.7) and 7.5 (6.7), respectively.

At 8 weeks (Table 2), there were significant group×time interaction effects for perceived stress and depressive symptoms; the treatment group experienced greater improvements from baseline to 8 weeks in both outcomes than the control group. For perceived stress, mean scores in the treatment group improved from 2.1 (SD=1.1) to 1.3 (SD=0.7), whereas scores in the control group worsened from 2.4 (SD=0.9) to 2.5 (SD=0.8) (−0.80 vs. +0.10; *p*<0.05). For depressive symptoms, the treatment group improved from 7.8 (SD=6.7) to 2.1 (SD=2.8), considered as remission of symptoms that were mild at baseline; however, in the control group, depressive symptoms improved slightly from 7.5 (SD=6.7) to 7.2 (SD=6.1) (−13.2 vs. −0.3; *p*<0.05).

**Table 2.** Perceived stress and depressive symptoms among Mentes Positivas en Acción participants: treatment group versus delayed intervention control group, and delayed intervention control group after receiving the Mentes Positivas en Acción program

Outcome measure	Treatment group compared with delayed-intervention control group					Delayed-intervention control group^a^			
	Baseline, mean (SD)		8-weeks, mean (SD)						
	Treatment (*N*=70)	Control (*N*=70)	Treatment (*N*=61)	Control (*N*=29)	*p*^b^	8-weeks (*N*=26), mean (SD)	16-weeks,^c^ (N=26), mean (SD)	Change^d^ (SD)	*p*
Perceived stress (PSS)	2.1 (1.1)	2.4 (0.9)	1.3 (0.7)	2.5 (0.8)	0.014	2.5 (0.8)	1.7 (0.7)	−0.8 (0.8)	<0.000
Depressive symptoms (PHQ-9)	7.8 (6.7)	7.5 (6.7)	2.1 (2.8)	7.2 (6.1)	0.011	7.2 (6.1)	3.3 (5.0)	−3.9 (5.5)	0.002

^a^ After an 8-week delayed-intervention control group waiting period, before beginning MPA program.

^b^ Group×time interaction.

^c^ After receiving MPA program.

^d^ Pre–post MPA program change score.

MPA, Mentes Positivas en Acción; PSS, Perceived Stress Scale.

Within-group changes in the control group demonstrated significant improvements in stress and depressive symptoms after completing the program (Table 2). Mean stress scores improved from 2.5 (SD=0.8) before the program to 1.7 (SD=0.7) after completing the program (−0.8; *p*<0.000). Mean depressive symptom scores improved from 7.2 (SD=6.1) to 3.3 (SD=5.0) (−3.9; *p*<0.002), considered as remission of symptoms that were mild at baseline.

## Discussion

In this study, we tested the feasibility and preliminary effectiveness of a community-based, promotor-delivered stress management program among Spanish-speaking Latino men and women. Compared with a delayed intervention control group, the immediate treatment group reported significant improvements on perceived stress and depressive symptoms. The results provide preliminary evidence of the effectiveness of MPA in reducing stress and depression among a high-risk vulnerable group of Latino immigrants.

Promotores delivered the program with excellent fidelity, demonstrating knowledge of stress and mood management techniques, consistent with other studies of promotor-delivered stress management programs.^20,31^ Our academic–community partnership with Monument Impact allowed us to access promotores and participants. We utilized this important community asset to house the project, while building its capacity to deliver a stress management program. We believe that this enhanced community trust in the program. To mitigate known challenges of recruitment, attendance, retention, and acceptability of behavioral programs among Latino immigrants, we emphasized community engagement methods for translating evidence-based interventions for underserved populations.^18,27^ Studies that engage the communities affected by health disparities are more likely to reach vulnerable populations.^20,22,32,33^

Community interest in MPA was enhanced by its being offered in Spanish, consistent with previous research.^30^ In spite of this, we experienced difficulties retaining participants, primarily among the control group during the 8-week waiting period for the intervention. One possible explanation is inactivation of cell phones, given that many immigrants have temporary cell phone plans, or change plans frequently. Another is that participants may not have answered the follow-up phone call attempts due to distrust of unknown telephone numbers in an anti-immigrant climate. Fear of deportation and a desire to remain hidden are common in research with this population,^34–36^ and this may have contributed to low retention.

Participants reported mild levels of stress and depression at baseline. Self-reporting of stress and depression symptoms among Latino immigrants is not well understood.^23^ Stigma is often associated with low reporting and utilization of behavioral services among low-income Latino.^37^

Study limitations include that this was a self-selected sample, as individuals had to contact Monument Impact to participate. Further, results may only generalize to Spanish-speaking Latinos of Mexican origin; however, the cultural elements of the intervention should apply to other Latino groups. We experienced differential attrition by experimental group, with a higher dropout rate in the control group, which could have introduced bias and overestimation of program effectiveness. Collecting data in future studies that could help shed light on barriers to attendance and engagement is advised.

Our study suggests that promotor-delivered programs such as MPA that support the mental health of Latinos may increase access to much needed services, especially among immigrant populations. Further, offering these services within trusted community organizations whose staff understand the cultural and social context for Latinos could help address their underutilization of mental health services. Further extensions of the program might include formation of clinic-community organization partnerships for further testing and dissemination of the program. In addition, testing the program among those with more severe symptoms of depression or anxiety is needed.

Further research on the promotor-delivered MPA program is needed to better understand contextual factors and conditions in real-world settings that contribute to better program uptake and positive outcomes. Future translational research could help identify any needed adaptations to the MPA program for use among Latinos with more severe symptoms of depression or anxiety, and its effectiveness in these populations. Translational research could also be conducted to investigate the relevance of the MPA program for other minority and immigrant populations.

## Conclusions

The study fills a gap in needed research that tests culturally congruent, evidence-based interventions delivered in the community to reach lower-income Latinos.^38^ Addressing barriers to access to culturally adapted stress management programs by training promotores is a promising approach.^39^ Vulnerable populations such as lower-income Latinos typically live in high-stress environments and have a limited sense of control. Acquisition of stress management skills constitutes a critical practical intervention. The mental health needs of a growing Latino population in the United States must be supported by community programs offered by promotores and other community practitioners that enhance skills for coping with stressful situations.^23^

### Future directions and sustainability

Implementation of this study sparked interest from local community health organizations, which recognize the need and disparities among Latinos accessing behavioral health programs. John Muir Health, a major local health care stakeholder, funded subsequent delivery of MPA by Monument Impact through John Muir Health's Community Benefit Programs. John Muir Health's role in sustaining MPA is driven by the organization's goals of overcoming barriers to mental health care in the Spanish-speaking community. Since this study was completed in 2017, MPA has been delivered by promotores to more than 600 participants in the community. John Muir's evaluation of the MPA (with no control or comparison group) in these participants has yielded similar results in terms of pre–post improvements in stress and depressive symptoms. Changes to the MPA program since this study include providing childcare for participants, program evaluation by participants, and attrition follow-up. Also, since the study was completed, referrals of individuals by community clinics to the MPA program have included individuals experiencing mild to severe stress and depressive symptoms. The growing number of Latinos in the United States and their limited access to culturally and linguistically congruent programs behooves finding feasible solutions and creative ways to ameliorate the behavioral health disparities that exist among Latinos and potentially other underserved at-risk groups.

## Abbreviations Used

MPA: Mentes Positivas en Acción
PI: Principal Investigator
PSS: Perceived Stress Scale
RA: Research Associate
SD: standard deviation
